# Artificial Intelligence-Based Quantitative Structure–Property Relationship Model for Predicting Human Intestinal Absorption of Compounds with Serotonergic Activity

**DOI:** 10.1021/acs.molpharmaceut.2c01117

**Published:** 2023-04-18

**Authors:** Natalia Czub, Jakub Szlęk, Adam Pacławski, Klaudia Klimończyk, Matteo Puccetti, Aleksander Mendyk

**Affiliations:** † Department of Pharmaceutical Technology and Biopharmaceutics, Jagiellonian University Medical College, 30-688 Kraków, Poland; ‡ Department of Pharmaceutical Sciences, University of Perugia, 06123 Perugia, Italy

**Keywords:** AI-based system, human intestinal absorption, QSPR, serotonergic activity, AutoML

## Abstract

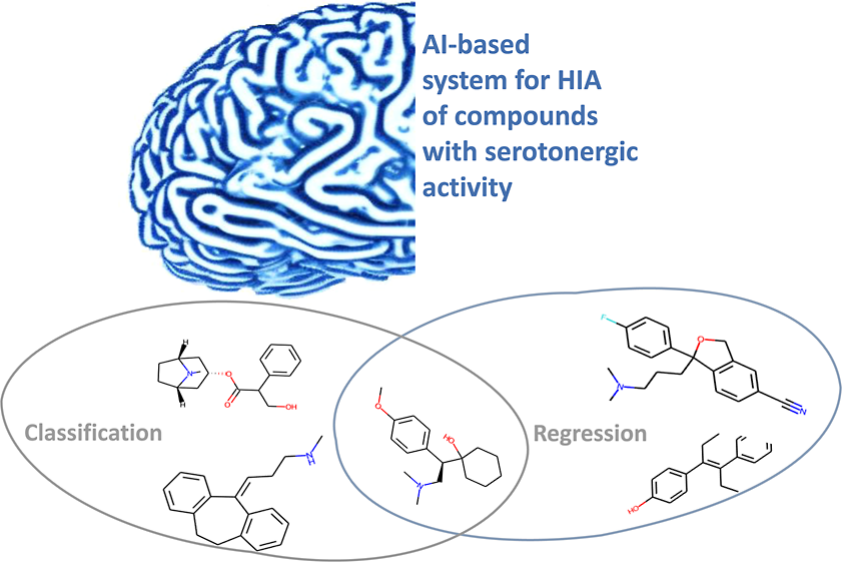

Oral medicines represent the largest pharmaceutical market area. To achieve a therapeutic effect, a drug must penetrate the intestinal walls, the main absorption site for orally delivered active pharmaceutical ingredients (APIs). Indeed, predicting drug absorption can facilitate candidate screening and reduce time to market. Algorithms are available with good prediction accuracy that however focus only on solubility. In this work, we focused on drug permeability looking at human intestinal absorption as a marker for intestinal bioavailability. Being of considerable therapeutic relevance, APIs with serotonergic activity were selected as a dataset. Due to process complexity, experimental data scarcity, and variability, we turned toward an artificial intelligence (AI)-based system, which is a hierarchical combination of classification and regression models. This combination of seemingly two models into a single system widens the space of molecules classified as highly permeable with high accuracy. The specialized and optimized system enables in silico and structure-based prediction with a high degree of certainty. Predictions in external validation allowed correct selection of the 38% of highly permeable molecules without any false positives. The proposed system based on AI represents a promising tool useful for oral drug screening at an early stage of drug discovery and development. Datasets and the obtained models are available on the GitHub platform (https://github.com/nczub/HIA_5-HT).

## 1 Introduction

The oral route of administration is the most preferred and natural way of drug dosing.^1^ Despite undisputed advantages, the biggest drawback of this route of administration is bioavailability, which may hinder the effect or even make it impossible for many compounds to be delivered by ingestion. Currently, all leading regulatory agencies like International Council on Harmonization (ICH), the Food and Drug Administration (FDA), and the European Medicines Agency (EMA) adopt the Biopharmaceutics Classification System (BCS)^2^ for sorting active pharmaceutical ingredients (APIs) based on water solubility and intestinal permeability. The BCS distinguishes four classes of APIs:

- Class I: high solubility, high permeability,
- Class II: low solubility, high permeability,
- Class III: high solubility, low permeability,
- Class IV: low solubility, low permeability.

In recent years, considerable effort has been spent to establish suitable tools to predict intestinal absorption with the aim of facilitating drug candidate screening at early stages of drug discovery and development. To this purpose, several algorithms have been proposed with good prediction accuracy, most of which though focusing only on solubility.^3−6^ A few other studies have been aimed at predicting intestinal permeability. For these reasons, issues concerning the second feature of BCS classes, which is solubility, was not the subject of the presented research. In this study, we focused on the permeability of the therapeutic substance through the intestinal wall. Good intestinal absorption is one of the major prerequisites to ensure APIs’ oral administration. The absorption limit, which defines the division of compounds into highly permeable and poorly permeable, is 85%.^7,8^ In vitro intestinal permeability studies are carried out using the following: parallel artificial membrane permeability assay (PAMPA), the human colorectal adenocarcinoma cell line (Caco-2), the Madin–Darby canine kidney (MDCK), and the porcine kidney epithelial cell lines (LLC-PK1). The Caco-2 cell line is the most employed owing to morphology and functionality closely resembling those of human enterocytes.^9^ For such reason, the use of Caco-2 cell line is recommended in the FDA and EMA guidelines that also define the threshold that discriminates between highly permeable and poorly permeable compounds, which is recognized as being the 85% of absorption.^7,8^

Membrane permeability is a property of compounds that can be predicted from structure using quantitative structure–property relationship (QSPR) models. These models can be obtained through machine learning (ML) methods. Based on the available dataset of molecules sorted by structure and properties, the algorithm learns to predict the dependent variable. Furthermore, the model can be used to predict a given feature for new compounds.^10−12^ Early papers on permeability prediction appeared in the 1990s proposing simple models based on linear, nonlinear, or partial least-squares regression. The number of compounds included was in the range of 6–35.^13−15^ Later, Castillo-Garit et al. developed a classification model based on 157 compounds belonging to structurally and pharmacologically diverse groups. Linear discriminant analysis was used to create the model based on TOMOCOMD-CARDD descriptors. The accuracy of this model was over 90 and 84% for the training and test dataset, respectively.^16^ In 2008, Yan et al. attempted to predict human intestinal absorption (HIA) using Support Vector Machine Regression. Molecular descriptors for more than 500 compounds were calculated using the ADRIANA Code. The best model had a coefficient of determination of *R*^2^ = 0.65 (training dataset) and *R*^2^ = 0.73 (testing dataset). The root-mean-square deviation for the entire model was 16.35.^17^ Sun et al. focused on permeability prediction through the intestinal wall expressed as *P*_eff_ (human effective intestinal membrane permeability). The model was developed based on 30 molecules, allocated into training and testing datasets in a 2:1 ratio. They used multiple linear regression and obtained *R*^2^ values of 0.76 and 0.86 for training and testing datasets, respectively.^18^ Another study published in 2016 used almost 1300 compounds and 2D molecular descriptors for model creation. The predicted value was log *P*_app_ (logarithm of apparent permeability). Multiple linear regression, partial least-squares regression, support vector machine, and boosting algorithms were used to create classification and regression QSPR models according to a 5-fold cross-validation. Metrics for the best regression model were RMSE (root-mean-square error) = 0.34 and *R*^2^ = 0.81 and for classification model with accuracy = 87.7% relative to the training dataset.^9^ Likewise, Wang et al. developed a QSPR model to predict log *P*_app_ on Caco-2 cells. Permeability data were collected from the literature and the ChEMBL database. 2D descriptors were calculated with PaDEL-descriptor software. The final database consisted in almost 1900 compounds and 261 descriptors. Models were created using six methods: MLR, SVR, XGBoost, RBF, dual-SVR, and Dual-RBF. The dual-RBF model suited the most providing *R*^2^ = 0.77 for the training dataset and *R*^2^ = 0.77 (5-CV) for the test dataset.^19^ Despite the large databases employed, the above works represented permeability through the intestinal wall using two rates of log *P*_app_ and *P*_eff_. Therefore, as supported by the literature,^20−22^ these values, being not highly correlated, are not directly representative of HIA.

In this work, to fill this gap, we narrowed down the compounds of interest to serotonergic drugs, an important drug class with a specific biological activity. Serotonin (5-hydroxytryptamine, 5-HT) is a biogenic monoamine that acts as a hormone, neurotransmitter, and mitogen in the central and peripheral nervous systems. It regulates nearly every aspect of human behavior.^23,24^ Serotonin receptors and transporters are important targets in the development of CNS drugs. Moreover, many existing drugs modulate serotonin neurotransmission. For example, anxiety-like behaviors are predominantly regulated by 5-HT_1A_ and 5-HT_2C_ receptors. However, the 5-HT_2C_ receptor regulates not just anxiety but also rewards processing, movement, hunger, and energy balance. Serotonin also influences several aspects of cardiac function, including electrical conduction, valve closure, and rebuilding after a heart attack.^25,26^

To the best of our knowledge, this study is the first to focus on compounds with serotonergic activity, and the only previous work focusing on HIA for a selected group of receptor family ligands concerned β-adrenoreceptor antagonists.^13^

For the above purpose, we employed an artificial intelligence (AI)-based approach for predicting HIA for serotonergic molecules. AI is a field that is increasingly used today. In drug discovery and development, it is also widely employed in areas such as the discovery of new lead structures, prediction of their activity against biological targets (QSAR models—quantitative structure–activity relationship), prediction of properties (QSPR), and the fate of the drug in the human body—ADME processes (absorption, distribution, metabolism, and excretion).^27−30^ The more AI develops, the newer terms have been created to describe new branches of this science. AI-based systems are functional software systems with at least one AI component. It is mostly used for speech or image recognition and autonomous cars.^31^ Based on the article from 2018, in these systems, the rules and behavior of the system are inferred from learning data rather than written as a program code.^32^ Being applied on a homogeneous dataset of serotonergic drugs, our approach can grant better correlation with actual HIA, so as to promote the development of useful tools for effective early screening of oral drug candidates.

## 2 Experimental Section

### 2.1 Database

We combined two starting datasets to give the database to be employed in this work. The first starting dataset was formed by the data collected from the literature and the ChEMBL database (April 2022)^33^ where permeability was expressed as HIA. Supporting Information Table 1 (SI1) displays the sources of compounds and their number, with 2314 molecules in total, from which 1207 unique molecules were extracted after removing duplicates based on SMILES (simplified molecular-input line-entry system). Supporting Information Table 2 (SI2) contains the database with the name/id of unique compounds, SMILES, HIA values, and source article with the title and DOI. The second starting dataset focused on serotonergic activity. Particular care was taken in selecting a curated database of serotonergic compounds with known intestinal absorption. In this study, in vitro serotonergic activity was not taken into account. A dataset of compounds with serotonergic activity was aggregated from the ZINC and ChEMBL databases^33,34^ (January 2022). The dataset remained with 31,323 unique compounds after removing duplicates. The final database was obtained by extracting compounds common to both datasets upon SMILES structure comparison. The final database resulted in 141 compounds for which bioavailability was obtained from literature reviewing.^17,35−42^ The data are summarized in Supporting Information Table 3 (SI3) (id, SMILES, and HIA values). The final database was then divided into training and test datasets in a ratio of 70:30. The database split was created using the train_test_split function from the Scikit-learn library (random_state = 42).^43^ The test dataset was used for external validation.

### 2.2 Descriptors

2D molecular descriptors calculated in Mordred software were used for molecular representation.^44^ After data preprocessing (mean imputation and constant input removal), 1241 input variables were obtained as the basis for the QSPR model creation.

### 2.3 QSPR Model

#### 2.3.1 Model Metrics

In this study, we have created two types of models: regression and binary classification. In the former, the dependent variable is a continuous value, while in the latter, it is a specific class. According to the FDA and EMA guidelines,^7,8^ as reported above, a molecule is classified as highly permeable when HIA ≥85% (class “1”) and poorly permeable if HIA <85% (class “0”). We find hereinafter the metrics for models’ evaluation.

##### 2.3.1.1 Regression

We used RMSE, normalized root-mean-square error (NRMSE), and coefficient of determination (R^2^) to evaluate the regression models. Equations 1–3 are shown below. The best model should have the lowest error and the highest *R*^2^.
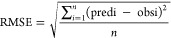
1where RMSE = root-mean-square error, obs_*i*_ and pred_*i*_ = observed and predicted values, *i* = data record number, and *n* = total number of records.
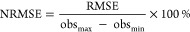
2where NRMSE = normalized root-mean-square error, RMSE = root-mean-square error calculated for the model, obs_max_ = maximum value of the observed results, and obs_min_ = minimum value of the observed results.
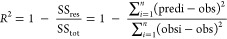
3where *R*^2^ = the coefficient of determination, SS_res_ = the sum of squares of the residual errors, SS_tot_ = the total sum of the errors, obs_*i*_ and pred_*i*_ = observed and predicted values, and obs = arithmetical mean of the observed values.

##### 2.3.1.2 Classification

Five metrics, namely, accuracy, precision, recall, cross-entropy loss (log loss), and F1, were employed to assess the classification models. They are presented below in eqs 4–8.
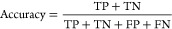
4
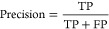
5
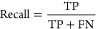
6where TP = true positive, TN = true negative, FP = false positive, and FN = false negative.
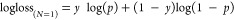
7where *y* = true label and *p* = probability estimate for class 1.
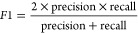
8In addition, we evaluated the models’ performance using a confusion matrix and the receiver operator characteristic curve (ROC curve). It is a statistic used to assess the performance of a model against all classification thresholds. A perfect model has AUC = 1, suggesting that its predictions are 100% correct, in contrast with models with AUC = 0, whose predictions are 100% wrong. Moreover, when AUC = 0.5, the model makes a random guess about predictions.^45,46^

#### 2.3.2 Model Development

The QSPR models were prepared using mljar^47^ according to a 10-fold cross-validation (10-CV). We carried out several experiments in which computation time was the differentiating factor (from 1 to 48 h). The number of trained and tested models for regression and classification was about 13,000 for each type.

## 3 Theoretical Basis

### 3.1 Mljar

Mljar is an automated ML (AutoML) tool designed to develop classification and regression models. At the same time, the tool adjusts the appropriate metrics evaluating the created model to the selected problem. Mljar provides numerous algorithms starting from simple baseline, where the predictions are either the most frequent class (in the case of classification) or the average of the output (in the case of regression), linear, through more complex, like decision trees, random forests, extra trees, LightGBM, XGBoost, CatBoost, neural networks, and nearest neighbors. Such algorithms can be applied to classification as well as regression problems. Usually, these methods use separate functions corresponding to a classification or regression problem. For example, the linear algorithm is divided into linear regression for regression and logistic classification for classification tasks. A typical classification algorithm includes the nearest neighbor and a decision tree-based method. However, mljar settings can be adjusted according to the type of the dependent variable; then, the program chooses the appropriate function, e.g., for random forest—random forest classifier (classification) and random forest regressor (regression). AutoML builds up a model from several smaller models (ensemble model) while tuning the model’s hyperparameters. The tool selects the best model among those created by itself.^47^

### 3.2 Model Explainability—Explainable AI

Model development and application to future and unknown event predictions is one important application, but the deployment of the knowledge accumulated and encoded in the model is equally fundamental. Understanding the way models generate prediction and interpretation is always embedded in the process of model development, starting from simple linear regression, where the model’s parameters quantify the impact and its direction on model prediction, to artificial neural network where a plethora of model parameters are interconnected, making simplified interpretation much more difficult. In the last case, various methods have been developed to estimate variable importance and its total influence on the final prediction, including sensitivity analysis, which is one of the first methods formalized and described in the literature.^48^ At the very beginning, the proposed methods allowed us to order variables according to their total impact on the final prediction, whereas their further development allowed scientists to find directional impact. Over the last few years, several methods for unraveling the model structure were developed and introduced, which represent the need for shaping a model into an intelligible form as well as estimating variables’ impact on the final prediction. Recently, different methods for model explanation have been applied: local interpretable model agnostic explanations (LIME) with its modification called GraphLIME, anchors, meaningful perturbations, layer-wise relevance propagation (LRP), deep Taylor decomposition (DTD), prediction difference analysis (PDA), testing with concept activation vectors (TCAV), explainable graph neural networks (XGNN), textual explanations of visual models and Shapley additive explanations (SHAP) with its modifications like asymmetric Shapley values (ASV), and Shapley flow.^49^ From the available methods, SHAP was applied to analyze the developed model due to its universal nature and possibility to apply to any predictive model, as well as its strong theoretical background rooted in cooperative game theory. Lloyd Shapley introduced the Shapley value concept in 1953, and he was awarded in 2012 the Nobel Memorial Prize in Economic Sciences for its discovery. Shapley, in his research, was looking for the answer to the question “How much would a player be willing to pay for participating in a game?” What in general will depend on the expected payoff for that particular player. The proposed Shapley value is a unique solution for that problem satisfying the efficiency, additivity, null player property, and symmetry.^50^ Translating this theory to the predictive modeling field, the model might be treated as a coalition of variables and predictions as a result of the “game”. Then, for every variable, Shapley value might be calculated,^51^ what is the direct answer on how to fairly split the “payout” (= the prediction) among all the features. An instance for computation can be an individual feature value, e.g., for tabular data or a group of feature values. In the provided analysis, values of features were taken directly from the database, which puts the results directly in the context of the problem. SHAP analysis was performed for the classification model, and predicted probability values were taken for variable importance calculations, which increased the resolution of the analysis.^52^

## 4 Results

### 4.1 Database

**Figure 1:**
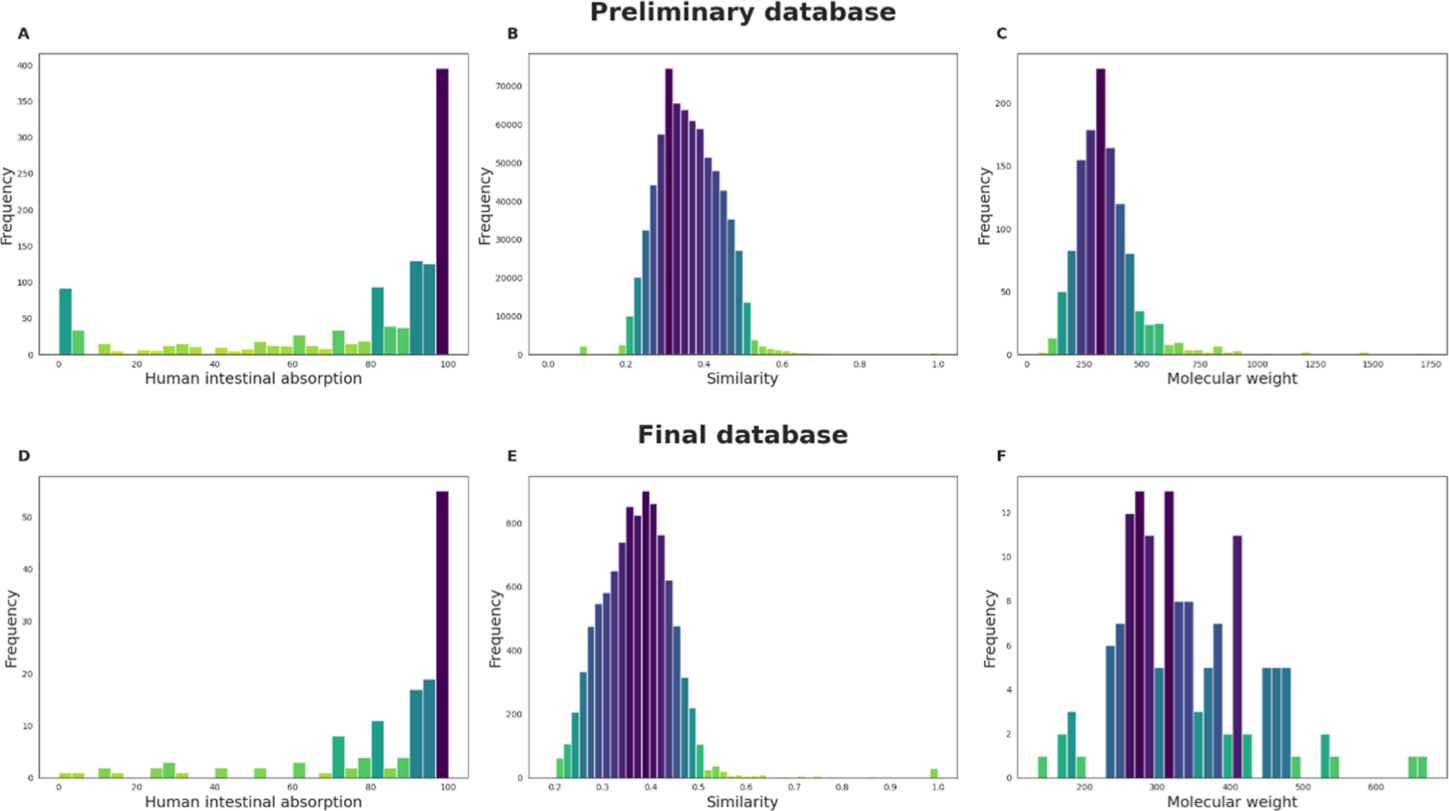
Distributions of the selected features for the starting and final databases: (A) HIA of the starting database. (B) Similarity of the starting database. (C) Molecular weight of the starting database. (D) HIA of the final database. (E) Similarity of the final database. (F) Molecular weight of the final database.

We generated a starting dataset of more than 1200 compounds and ended up with a final database of 141 serotonin compounds. Three discriminating features were accounted: HIA, compounds’ structural similarity, and molecular weight. The results are shown in Figure 1. It can be observed that HIA values are divided into two categories: high absorption (above 85%) and low absorption (below 85%). For the starting database, an additional area depicting HIA <10% is also highlighted. To assess the similarity of the compounds, we used the Tanimoto coefficient for each molecule pair. This coefficient establishes a similarity rank between 0 and 1, being 1 for identical molecules and 0 for lack of similarity.^53^ The coefficient ranged from 0 to 1 for the starting database, with a median of 0.357, while between 0.185 and 1 for the final database of 141 compounds, with a median of 0.372. Such similar medians and the wide range of values indicate low compound similarity for both databases. The molecular weight for the final database was between 135.21 and 670.85 (median = 314.86) whereas between 46.07 and 1736.18 (median = 323.59) for the starting database. The diversity of compounds suggests a vast applicability domain for the prediction model being developed.

### 4.2 QSPR Models

**Figure 2:**
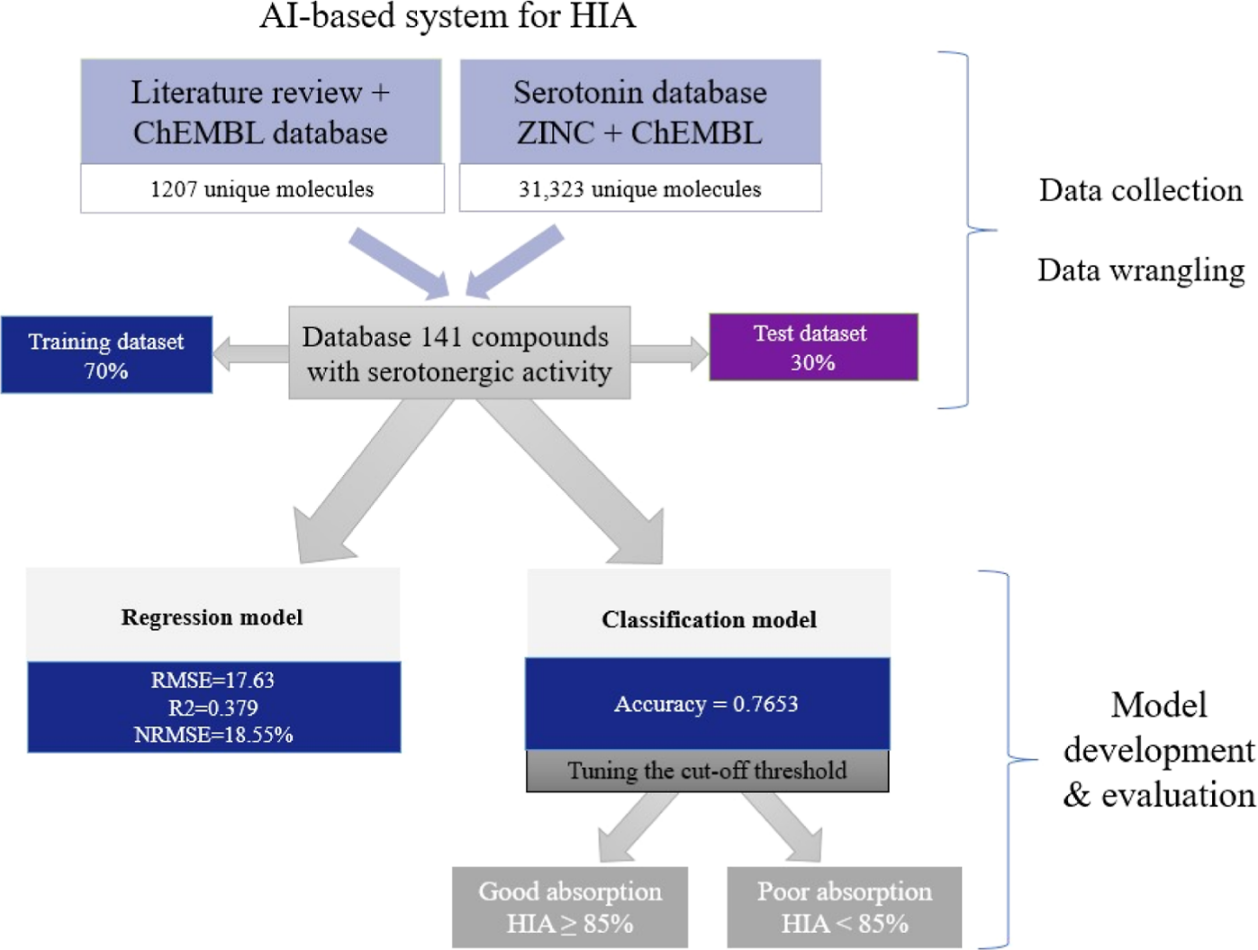
Methodology flow chart according to the 10-fold cross-validation of the AI-based system HIA model.

To clarify the complex multi-stage methodology adopted, the phases of model development and results according to 10-CV are summarized in Figure 2. The datasets and the obtained QSPR models are available on the GitHub platform (https://github.com/nczub/HIA_5-HT).

#### 4.2.1 Regression Model

At first, a regression model was developed to predict HIA for the final database of 141 serotonergic compounds. According to the above scheme, the model was created using a training dataset of 98 compounds according to 10-CV and validated using an external dataset of 43 compounds.

The best model obtained for the training dataset had RMSE = 17.63, NRMSE = 18.55%, and *R*^2^ = 0.379 and RMSE = 23.61, NRMSE = 23.61%, and *R*^2^ = 0.047 for the test dataset. The ensemble model was formed by five random forests and two neural networks. The obtained results indicate a high prediction error for both datasets. This low model performance was explained by the sparse HIA value distribution.

#### 4.2.2 Classification Model

**Figure 3:**
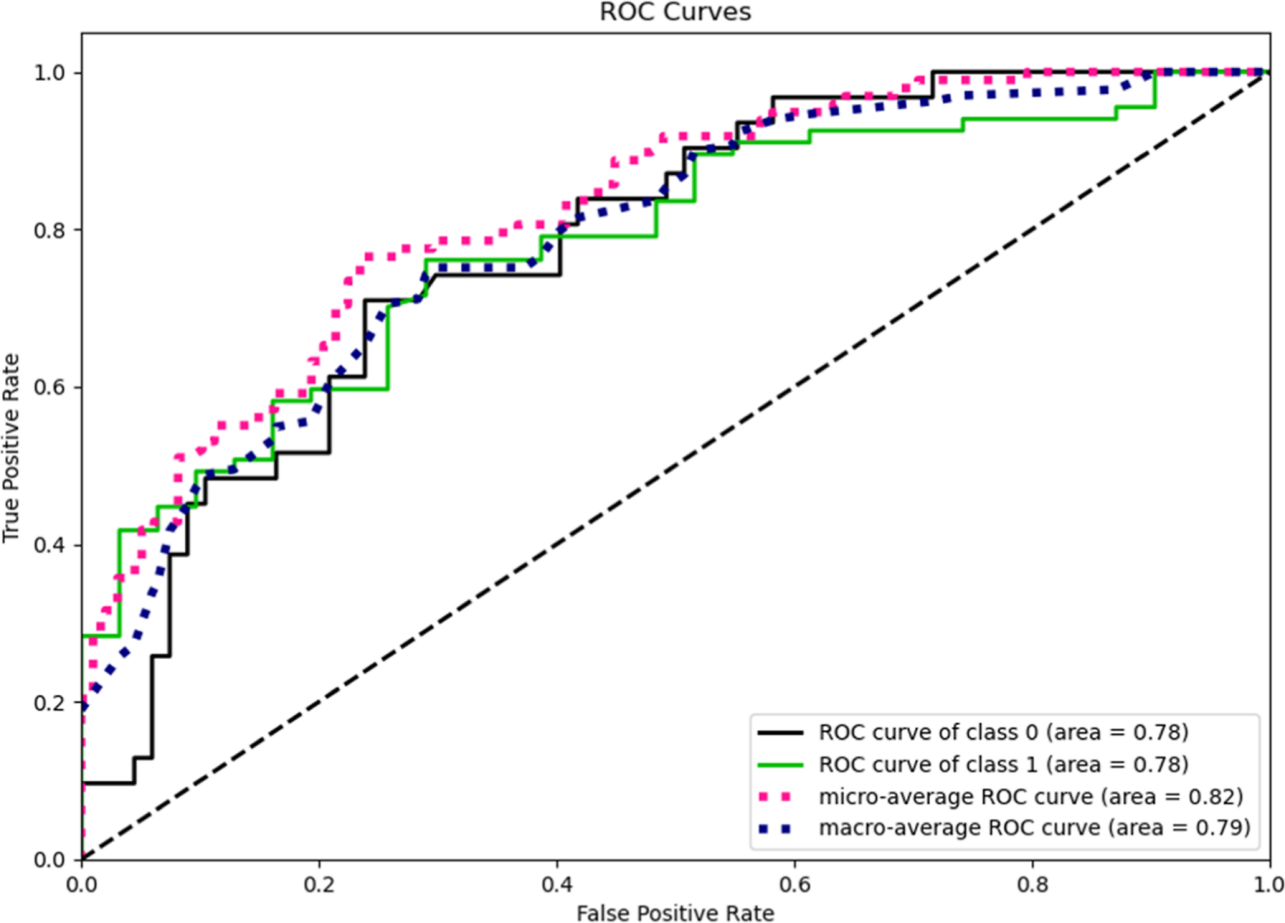
Four curves: (1) ROC curve for class 0—poorly permeable compounds. (2) ROC curve for class 1—highly permeable compounds. (3) Micro-average ROC curve. (4) Macro-average ROC curve, which is a plot of relation of the true positive rate against the false-positive rate.

**Figure 4:**
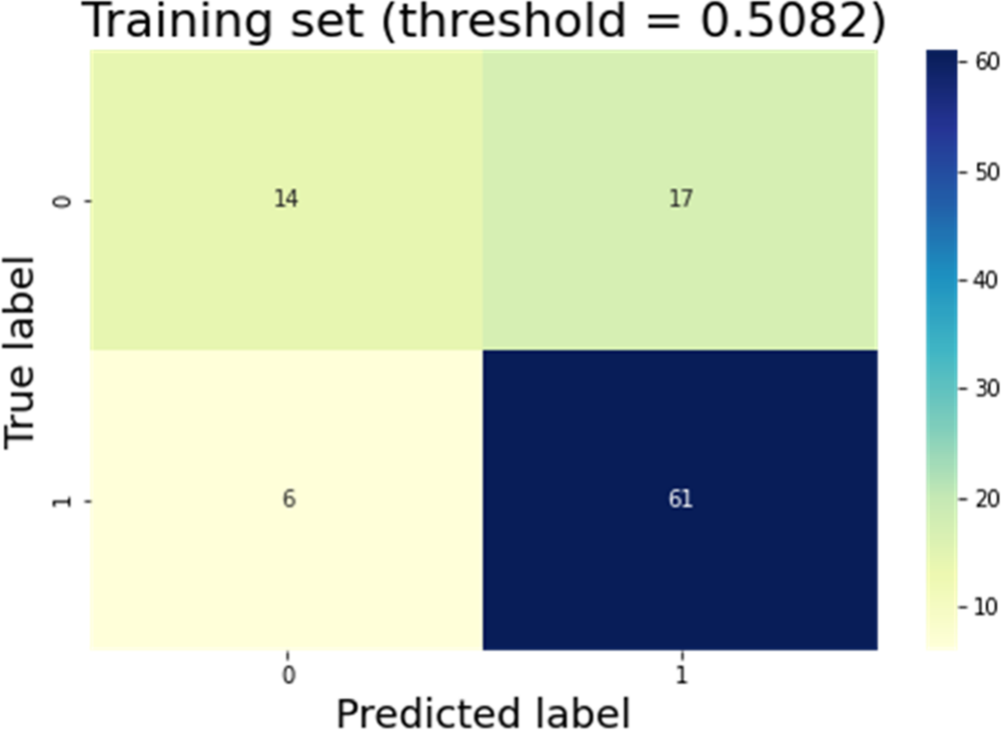
Confusion matrix of the training dataset.

**Figure 5:**
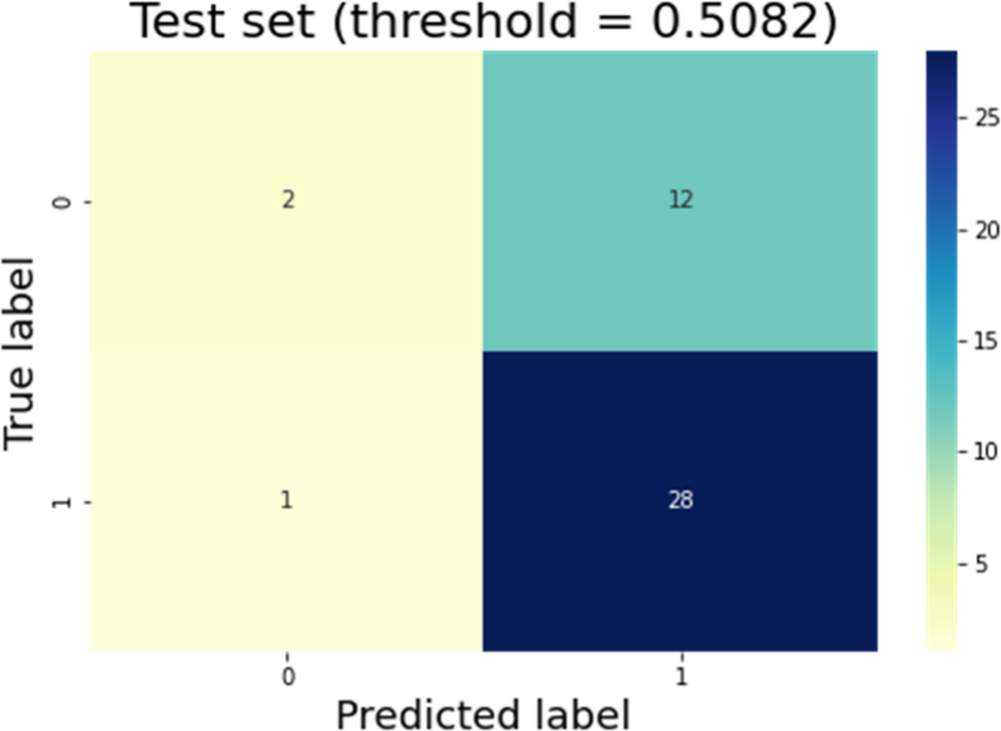
Confusion matrix of the test dataset.

Due to the poor performance of the regression model over the entire collection of compounds with serotonergic activity, we resorted to a binary classification model. By dividing the training and test datasets into the corresponding classes, we sorted 67 and 29 highly permeable and 31 and 14 poorly permeable compounds, respectively. The classification model obtained using mljar was an ensemble of seven smaller models (three CatBoosts, two neural networks, one extra tree, and one random forest). The results of metrics according to a 10-fold cross-validation and an external validation are shown in Table 1. The AutoML tool determined the level of probability for class 1 that classifies compounds as highly permeable with a probability larger than 0.5082. Figure 3 displays the ROC curves for class 0 and 1 and micro- and macro-average ROC curves. Figures 4 and 5 show the confusion matrix results for the training and test datasets with the original class selection truncation threshold.

**Table 1:** Metric Details for the Classification Model at Threshold = 0.5082

metrics	10-CV	test dataset
log loss	0.5102	0.5671
AUC	0.7826	0.7229
f1	0.8414	0.8116
accuracy	0.7653	0.6977
precision	0.7821	0.7000
recall	0.9104	0.9655

The model whose threshold was chosen automatically (threshold = 0.5082) had high false-positive rates [(17/31 = 0.548) and (12/14 = 0.857)] in the training and test datasets, respectively. These false positives are compounds incorrectly assigned to class 1 when their true class is the group of poorly permeable compounds. Table 2 reports the steps for tuning the cutoff for a highly permeable compound classifier.

**Table 2:** Tuning the Cutoff Threshold and Results According to 10-CV^a^

threshold	precision	true positive	false positive	true positive rate
0.5082	0.782	61	17	0.910
0.525	0.779	60	17	0.897
0.55	0.779	60	17	0.896
0.575	0.787	55	15	0.821
0.6	0.779	53	15	0.791
0.625	0.845	49	9	0.731
0.65	0.842	48	9	0.716
0.675	0.849	45	8	0.672
0.7	0.851	40	7	0.597
0.725	0.884	38	5	0.568
0.75	0.909	30	3	0.448
**0.775**	**0.964**	**27**	**1**	**0.403**
0.8	0.962	25	1	0.373
0.825	1.0	19	0	0.285
0.85	1.0	16	0	0.239
0.875	1.0	12	0	0.179
0.9	1.0	7	0	0.104

a The cutoff threshold was established based on the simultaneous satisfaction of three conditions: highest precision, lowest number of false positives, and minimum threshold value, without any alteration of the highly permeable compounds class. By this approach, a threshold value of 0.775 was achieved with a precision of 0.964 for the training dataset.

### 4.3 Case Study

We challenged our AI-based system for HIA prediction with the test dataset of compounds exhibiting serotonergic activity. The test dataset contained 29 highly permeable and 14 poorly permeable compounds. These compounds mimic a real-life scenario, which is a dataset of lead structures with serotonergic activity, which would be selected during the screening phase for serotonergic activity. The AI-based system is designed to suggest the user with a high level of confidence whether the compound will easily penetrate the intestinal wall.

**Figure 6:**
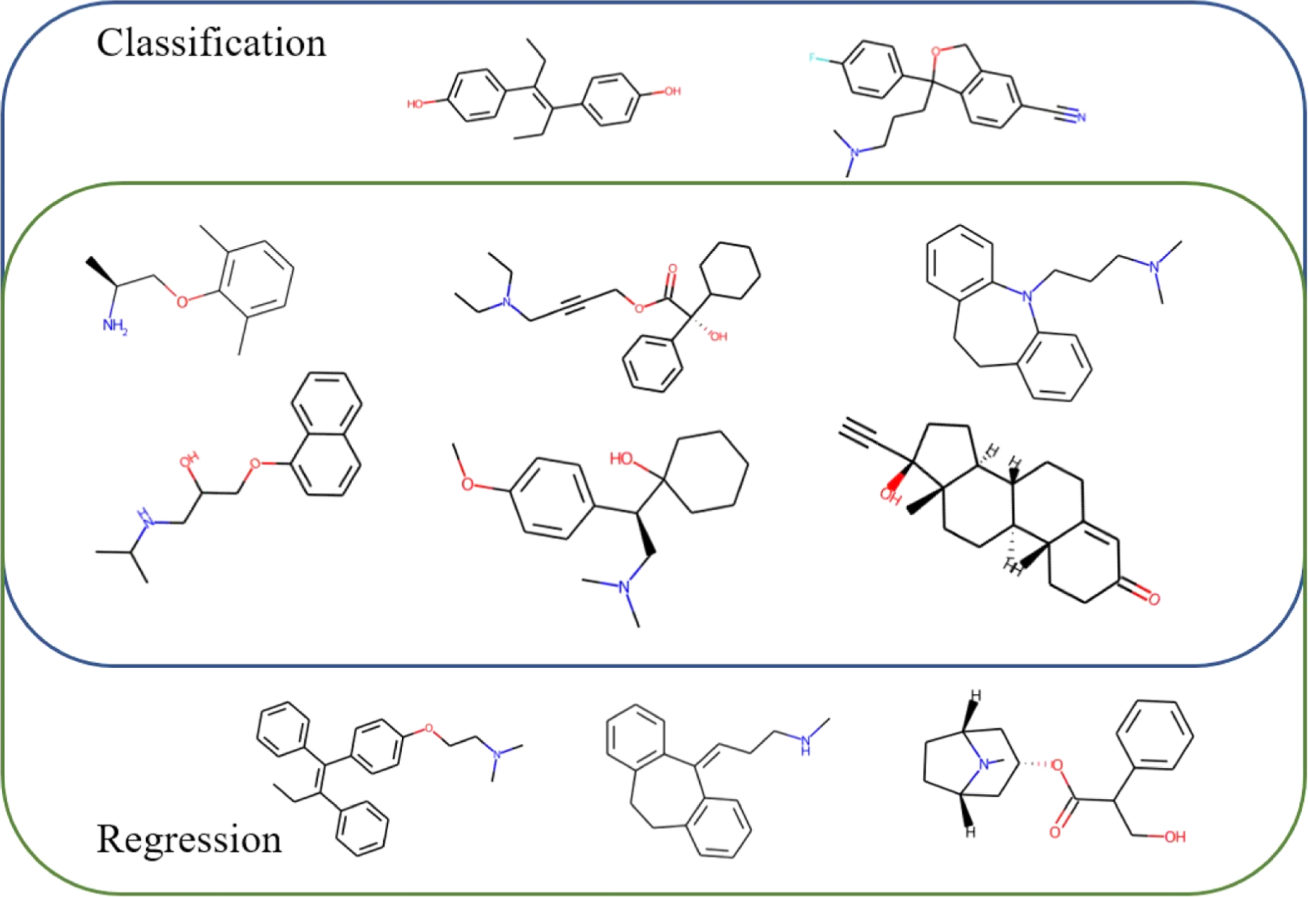
Compounds classified as highly permeable by the classification and regression models.

The system is based on two AutoML QSPR models. In the first step, a classification model with an increased cutoff threshold is used to extract compounds belonging to the class of highly permeable compounds with high confidence. With this model, eight compounds were correctly classified as class 1 with zero false positives. However, such a highly specialized model leads to the loss of highly permeable compounds. Therefore, in the second step, for the remaining compounds, we used a regression model also with an increased cutoff. In the classification model, the threshold between highly permeable and poorly permeable compounds was HIA = 85%. In the regression model, in order to achieve high prediction reliability, it was increased to ≥90%. With this model, an almost 40% increase in the number of highly bioavailable compounds was achieved. In total, the AI-based system qualified 11 compounds as highly permeable with no false-positive compounds for the test dataset. The outcome of the system is shown in Figure 6, where compounds classified as highly permeable by the classification and regression models are displayed. The system successfully identified 38% of highly permeable molecules. Such system shows a great potential in HIA screening application.

### 4.4 Shapley Additive Explanations

The classification model was analyzed to calculate the marginal impact of each variable on final predictions. For calculations, the training dataset was applied to reflect the real model learning environment without extrapolation. This might be important to understand how predictions are calculated considering the later performance analysis and the best-performing model pick. The range of |avSHAP| values, for 10 most important variables (Table 3), was 0.00923–0.00440. Being the range of model prediction probability 0.9224–0.2596, these variables cover approximately 10% of such space.

**Table 3:** Calculated |avSHAP| Values for the 10 Most Important Variables According to SHAP Analysis

variable	|avSHAP| value	description
MATS1dv	0.00923	Moran coefficient of lag 1 weighted by valence electrons
PEOE VSA7	0.00879	MOE charge VSA descriptor 7
MATS4s	0.00666	Moran coefficient of lag 4 weighted by intrinsic state
RPCG	0.00627	relative positive charge
AATSC4p	0.00611	averaged and centered Moreau–Broto autocorrelation of lag 4 weighted by polarizability
AMID X	0.00606	averaged molecular ID on halogen atoms
ATSC6i	0.00606	centered Moreau–Broto autocorrelation of lag 6 weighted by ionization potential
AATSC3d	0.00494	averaged and centered Moreau–Broto autocorrelation of lag 3 weighted by sigma electrons
MID N	0.00442	molecular ID on N atoms
GATS4i	0.00440	Geary coefficient of lag 4 weighted by ionization potential

**Figure 7:**
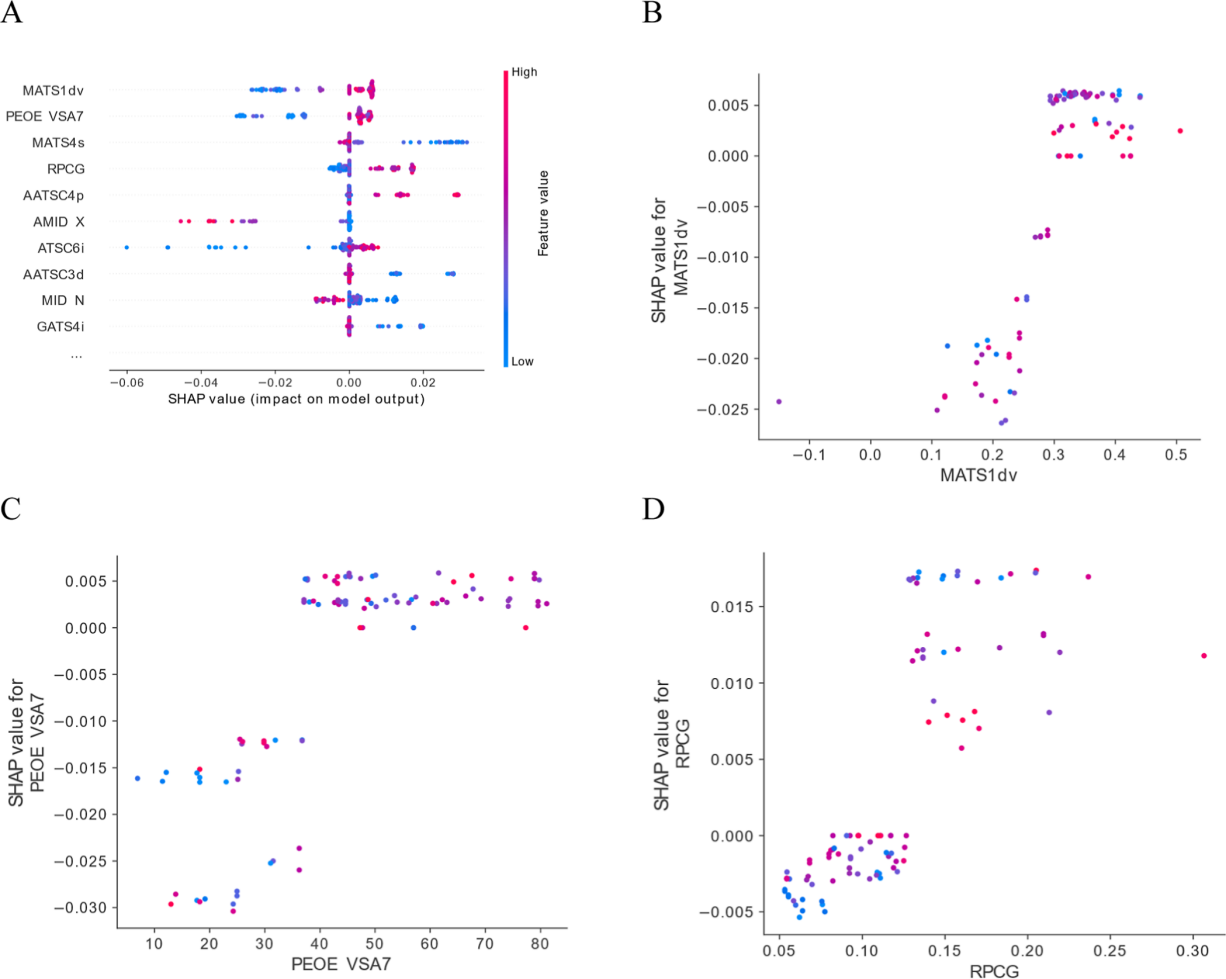
Results of SHAP analysis for the classification model. (A) Summary of the 10 most important variables represented in the graphical form. The single dot represents one record taken for analysis, whereas its color indicates the variable’s numerical value in the specific prediction (game). (B,C,D) Graphs represent the relation between the chosen variables’ numerical values and calculated participation in model’s prediction in SHAP analysis. Dot’s color represents the value of input features with highest correlation to the analyzed one in order to appreciate possible bias of the observed effect by another variable due to multicollinearity.

SHAP analysis delivers information not only about the global impact of features on the model predicted outcome. In fact, during computations, the marginal contribution is calculated for every presented input vector and a more in-depth analysis is allowed, such as the relation between the variable value and its impact on the forecast. A graphical representation summarizing the 10 most important variables and their functional relationship SHAP (variable) is described in Figure 7A. More detailed views on MATS1dv, PEOE VSA7, and RPCG are presented in Figure 7B–D, respectively. For all variables, it is observed that, within their value domain, some areas with positive, negative, or neutral impact might be distinguished. In the case of the Moran coefficient of lag 1 weighed by valence electrons, a value above 0.3 might be accounted as neutral or slightly positive, thus corresponding to a class with good intestine permeability, whereas a value below 0.3 reduces the probability of classification as a highly permeable molecule.

## 5 Discussion

Drug absorption in the intestine might be simplified into two processes, partition and diffusion.^54^ Both processes are still not fully understood in terms of molecule structure and process flow. Therefore, the assessment or classification of a molecule to the highly permeable class based only on the structure and without additional experiments is not possible with reasonable accuracy. Abraham et al. presented a few empirical equations in the form of linear regression models describing both processes affecting HIA. Due to the lack of external test predictions and additional mathematical operations performed for flattening model response by logarithm transformation, direct comparison of the results is troublesome. It is worth pointing out that the model’s analysis performed to elucidate its prediction structure shows the high influence of electric molecular properties on the classification results. Being absorption a mix of diffusion and partition processes, molecule polarizability might correlate with chances of weak interactions with cell membrane components. Moreover, charge-related molecular features can affect the partition process as well. The above description is related to the way the model prediction is calculated, taking into account the empirical character of the model, provided that hypotheses might require verification.

In this work, we addressed HIA for an important group of compounds that interact with serotonin receptors and serotonin transporters. A tool to predict permeability through the intestinal wall may help to assess whether a serotonergic candidate, namely, a potential drug for diseases such as depression, anxiety disorders, or schizophrenia, could manifest issues when orally delivered. Therefore, we propose a powerful support to the screening of such compounds at early drug discovery stage, wishing a rapid expansion of AI approaches to other drug classes. Generalization of our approach is not desirable as much as the application of AI models across too heterogeneous molecule datasets as often attempted in the available literature. It rather may be more useful to tailor particular systems to specific and more homogeneous molecule datasets.

## 6 Conclusions

The results of the starting regression model give a measure of the effort required to cope with such a difficult task. The level of prediction error makes it impossible to predict HIA values with acceptable accuracy. Thanks to AI-based systems, we decided to take a more comprehensive approach by creating a prediction pathway for the dataset. In the first step, a given compound is subjected to classification with a trimmed threshold. When the ligand is determined to be a highly permeable compound, then the response is highly correlated with real conditions. On the other hand, when a molecule is assigned to a low permeability class, a regression model with an increased prediction range will allow more serotonergic compounds to be classified as highly permeable.

In this project, we wanted to show how to deal with difficult data, which includes most biological data (small amount of data and distribution of data dominating one side of the value range). To our knowledge, this is the first study to involve two types of models as a prediction pathway (regression and classification) and an AI-based approach to predict HIA. The next step of this project will be to develop software to support the user in HIA prediction for serotonergic drug candidates.

## Supporting Information Available

Sources of compounds with HIA from the literature review and the ChEMBL database and their number (PDF)
Database with the name/id of unique compounds, SMILES, HIA values, and source article with the title and DOI number (XLSX)
Final database of 141 compounds’ id, SMILES, and HIA values (XLSX)

## Abbreviations

10-CV: 10-fold cross-validation
5-HT: 5-hydroxytryptamine, serotonin
5-HT1A: serotonin 1A receptor
5-HT2C: serotonin 2C receptor
AATSC3d: averaged and centered Moreau–Broto autocorrelation of lag 3 weighted by sigma electrons
AATSC4p: averaged and centered Moreau–Broto autocorrelation of lag 4 weighted by polarizability
ADME: absorption, distribution, metabolism, and excretion
AI: artificial intelligence
AMID X: averaged molecular ID on halogen atoms
API: active pharmaceutical ingredient
ASV: asymmetric Shapley values
ATSC6i: centered Moreau–Broto autocorrelation of lag 6 weighted by ionization potential
AUC: area under the curve
AutoML: automated machine learning
BCS: biopharmaceutics classification system
Caco-2: human colorectal adenocarcinoma cell line
DOI: digital object identifier
DTD: deep Taylor decomposition
EMA: European Medicines Agency
FDA: Food and Drug Administration
FN: false negative
FP: false positive
GATS4i: Geary coefficient of lag 4 weighted by ionization potential
HIA: human intestinal absorption
ICH: International Council on Harmonization
LightGBM: light gradient-boosting machine
LIME: local interpretable model agnostic explanations
LLC-PK1: porcine kidney epithelial cell line
log *P*_app_: logarithm of apparent permeability
LRP: layer-wise relevance propagation
MATS1dv,: Moran coefficient of lag 1 weighted by valence electrons
MATS4s: Moran coefficient of lag 4 weighted by intrinsic state
MDCK: Madin–Darby canine kidney
MID N: molecular ID on N atoms
ML: machine learning
MLR: multiple linear regression
NRMSE: normalized root-mean-square error
PAMPA: parallel artificial membrane permeability assay
PDA: prediction difference analysis
*P*_eff_: human effective intestinal membrane permeability
PEOE VSA7: MOE charge VSA descriptor 7
QSAR: quantitative structure–activity relationship
QSPR: quantitative structure–property relationship
*R*^2^: coefficient of determination
RBF: radial basis functions
RMSE: root-mean-square error
ROC curve: receiver operator characteristic curve
RPCG: relative positive charge
SHAP: Shapley additive explanations
SMILES: simplified molecular-input line-entry system
SVR: support vector regression
TCAV: testing with concept activation vectors
TN: true negative
TP: true positive
xAI: explainable artificial intelligence
XGBoost: extreme gradient boosting
XGNN: explainable graph neural networks

## References

[ref1] Abuhelwa A. Y.; Williams D. B.; Upton R. N.; Foster D. J. Food, gastrointestinal pH, and models of oral drug absorption. Eur. J. Pharm. Biopharm. 2017, 112, 234–248. 10.1016/j.ejpb.2016.11.034. 27914234

[ref2] Amidon G. L.; Lennernäs H.; Shah V. P.; Crison J. R. A Theoretical Basis for a Biopharmaceutic Drug Classification: The Correlation of in Vitro Drug Product Dissolution and in Vivo Bioavailability. Pharm. Res. 1995, 12, 413–420. 10.1023/A:1016212804288. 7617530

[ref3] Li M.; Chen H.; Zhang H.; Zeng M.; Chen B.; Guan L. Prediction of the Aqueous Solubility of Compounds Based on Light Gradient Boosting Machines with Molecular Fingerprints and the Cuckoo Search Algorithm. ACS Omega 2022, 7, 42027–42035. 10.1021/acsomega.2c03885. 36440111 PMC9685740

[ref4] Meng J.; Chen P.; Wahib M.; Yang M.; Zheng L.; Wei Y.; Feng S.; Liu W. Boosting the predictive performance with aqueous solubility dataset curation. Sci. Data 2022, 9, 71 10.1038/s41597-022-01154-3. 35241693 PMC8894363

[ref5] Francoeur P. G.; Koes D. R. SolTranNet-A Machine Learning Tool for Fast Aqueous Solubility Prediction. J. Chem. Inf. Model. 2021, 61, 2530–2536. 10.1021/acs.jcim.1c00331. 34038123 PMC8900744

[ref6] Bagheri M.; Golbraikh A. Rank-based ant system method for non-linear QSPR analysis: QSPR studies of the solubility parameter. SAR QSAR Environ. Res. 2012, 23, 59–86. 10.1080/1062936X.2011.623356. 22040297

[ref7] M9 Biopharmaceutics Classification System-Based Biowaivers, version 05.11.2021. https://www.fda.gov/regulatory-information/search-fda-guidance-documents/m9-biopharmaceutics-classification-system-based-biowaivers (accessed April 15, 2022).

[ref8] ICH M9 on Biopharmaceutics Classification System Based Biowaivers, version 11.02.2020. https://www.ema.europa.eu/en/ich-m9-biopharmaceutics-classification-system-based-biowaivers#current-version-section (accessed April 15, 2022).

[ref9] Wang N. N.; Dong J.; Deng Y. H.; Zhu M. F.; Wen M.; Yao Z. J.; Lu A. P.; Wang J. B.; Cao D. S. ADME Properties Evaluation in Drug Discovery: Prediction of Caco-2 Cell Permeability Using a Combination of NSGA-II and Boosting. J. Chem. Inf. Model. 2016, 56, 763–773. 10.1021/acs.jcim.5b00642. 27018227

[ref10] Liu R.; So S. S. Development of quantitative structure-property relationship models for early ADME evaluation in drug discovery. 1. Aqueous solubility. J. Chem. Inf. Comput. Sci. 2001, 41, 1633–1639. 10.1021/ci010289j. 11749590

[ref11] Clark R. D.; Daga P. R. Building a Quantitative Structure-Property Relationship (QSPR) Model. Methods Mol. Biol. 2019, 1939, 139 10.1007/978-1-4939-9089-4_8. 30848460

[ref12] Nguyen T. H.; Nguyen L. H.; Truong T. N. Application of Machine Learning in Developing Quantitative Structure-Property Relationship for Electronic Properties of Polyaromatic Compounds. ACS Omega 2022, 7, 22879–22888. 10.1021/acsomega.2c02650. 35811887 PMC9261278

[ref13] Palm K.; Luthman K.; Unge A. L.; Strandlund G.; Artursson P. Correlation of drug absorption with molecular surface properties. J. Pharm. Sci. 1996, 85, 32–39. 10.1021/js950285r. 8926580

[ref14] Norinder U.; Osterberg T.; Artursson P. Theoretical calculation and prediction of Caco-2 cell permeability using MolSurf parametrization and PLS statistics. Pharm. Res. 1997, 14, 1786–1791. 10.1023/a:1012196216736. 9453069

[ref15] Camenisch G.; Alsenz J.; van de Waterbeemd H.; Folkers G. Estimation of permeability by passive diffusion through Caco-2 cell monolayers using the drugs’ lipophilicity and molecular weight. Eur. J. Pharm. Sci. 1998, 6, 313–319. 10.1016/s0928-0987(97)10019-7. 9795088

[ref16] Castillo-Garit J. A.; Marrero-Ponce Y.; Torrens F.; García-Domenech R. Estimation of ADME properties in drug discovery: predicting Caco-2 cell permeability using atom-based stochastic and non-stochastic linear indices. J. Pharm. Sci. 2008, 97, 1946–1976. 10.1002/jps.21122. 17724669

[ref17] Yan A.; Wang Z.; Cai Z. Prediction of Human Intestinal Absorption by GA Feature Selection and Support Vector Machine Regression. Int. J. Mol. Sci. 2008, 9, 1961–1976. 10.3390/ijms9101961. 19325729 PMC2635609

[ref18] Sun L.; Liu X.; Xiang R.; Wu C.; Wang Y.; Sun Y.; Sun J.; He Z. Structure-based prediction of human intestinal membrane permeability for rapid in silico BCS classification. Biopharm. Drug Dispos. 2013, 34, 321–335. 10.1002/bdd.1848. 23716273

[ref19] Wang Y.; Chen X. QSPR model for Caco-2 cell permeability prediction using a combination of HQPSO and dual-RBF neural network. RSC Adv. 2020, 10, 42938–42952. 10.1039/D0RA08209K. 35514900 PMC9058322

[ref20] Larregieu C. A.; Benet L. Z. Distinguishing between the permeability relationships with absorption and metabolism to improve BCS and BDDCS predictions in early drug discovery. Mol. Pharm. 2014, 11, 1335–1344. 10.1021/mp4007858. 24628254 PMC3983369

[ref21] Dahlgren D.; Roos C.; Sjögren E.; Lennernäs H. Direct In Vivo Human Intestinal Permeability (Peff) Determined with Different Clinical Perfusion and Intubation Methods. J. Pharm. Sci. 2015, 104, 2702–2726. 10.1002/jps.24258. 25410736

[ref22] Thomas S.; Brightman F.; Gill H.; Lee S.; Pufong B. Simulation modelling of human intestinal absorption using Caco-2 permeability and kinetic solubility data for early drug discovery. J. Pharm. Sci. 2008, 97, 4557–4574. 10.1002/jps.21305. 18300298

[ref23] Mohammad-Zadeh L. F.; Moses L.; Gwaltney-Brant S. M. Serotonin: a review. J. Vet. Pharmacol. Ther. 2008, 31, 187–199. 10.1111/j.1365-2885.2008.00944.x. 18471139

[ref24] Sarrouilhe D.; Defamie N.; Mesnil M. Is the Exposome Involved in Brain Disorders through the Serotoninergic System?. Biomedicines 2021, 9, 1351 10.3390/biomedicines9101351. 34680468 PMC8533279

[ref25] Berger M.; Gray J. A.; Roth B. L. The Expanded Biology of Serotonin. Annu. Rev. Med. 2009, 60, 355–366. 10.1146/annurev.med.60.042307.110802. 19630576 PMC5864293

[ref26] Doggrell S. The role of 5-HT on the cardiovascular and renal systems and the clinical potential of 5-HT modulation. Expert Opin. Invest. Drugs 2003, 12, 805–823. 10.1517/13543784.12.5.805. 12720492

[ref27] Hessler G.; Baringhaus K. H. Artificial Intelligence in Drug Design. Molecules 2018, 23, 2520 10.3390/molecules23102520. 30279331 PMC6222615

[ref28] Basile A. O.; Yahi A.; Tatonetti N. P. Artificial Intelligence for Drug Toxicity and Safety. TIPS 2019, 40, 624–635. 10.1016/j.tips.2019.07.005. 31383376 PMC6710127

[ref29] Maltarollo V. G.; Gertrudes J. C.; Oliveira P. R.; Honorio K. M. Applying machine learning techniques for ADME-Tox prediction: a review. Expert Opin. Drug Metab. Toxicol. 2015, 11, 259–271. 10.1517/17425255.2015.980814. 25440524

[ref30] Tao L.; Zhang P.; Qin C.; Chen S. Y.; Zhang C.; Chen Z.; Zhu F.; Yang S. Y.; Wei Y. Q.; Chen Y. Z. Recent progresses in the exploration of machine learning methods as in-silico ADME prediction tools. Adv. Drug Delivery Rev. 2015, 86, 83–100. 10.1016/j.addr.2015.03.014. 26037068

[ref31] Martínez-Fernández S.; Bogner J.; Franch X.; Oriol M.; Siebert J.; Trendowicz A.; Vollmer A. M.; Wagner S. Software Engineering for AI-Based Systems: A Survey. ACM Trans. Softw. Eng. Methodol. 2022, 31, 1–59. 10.1145/3487043.

[ref32] Khomh F.; Adams B.; Cheng J.; Fokaefs M.; Antoniol G. Software engineering for machine-learning applications: The road ahead. IEEE Software 2018, 35, 81–84. 10.1109/MS.2018.3571224.

[ref33] Mendez D.; Gaulton A.; Bento A. P.; Chambers J.; de Veij M.; Félix E.; Magariños M. P.; Mosquera J. F.; Mutowo P.; Nowotka M.; et al. ChEMBL: Towards direct deposition of bioassay data. Nucleic Acids Res. 2018, 47, D930–D940. 10.1093/nar/gky1075. PMC6323927 30398643

[ref34] Irwin J. J.; Shoichet B. K. ZINC--a free database of commercially available compounds for virtual screening. J. Chem. Inf. Model. 2005, 45, 177–182. 10.1021/ci049714+. 15667143 PMC1360656

[ref35] Balon K.; Riebesehl B. U.; Müller B. W. Drug liposome partitioning as a tool for the prediction of human passive intestinal absorption. Pharm. Res. 1999, 16, 882–888. 10.1023/a:1018882221008. 10397609

[ref36] Zhao Y. H.; Abraham M. H.; Le J.; Hersey A.; Luscombe C. N.; Beck G.; Sherborne B.; Cooper I. Rate-limited steps of human oral absorption and QSAR studies. Pharm. Res. 2002, 19, 1446–1457. 10.1023/a:1020444330011. 12425461

[ref37] Klopman G.; Stefan L. R.; Saiakhov R. D. ADME evaluation. Eur. J. Pharm. Sci. 2002, 17, 253–263. 10.1016/s0928-0987(02)00219-1. 12453615

[ref38] Varma M. V.; Sateesh K.; Panchagnula R. Functional role of P-glycoprotein in limiting intestinal absorption of drugs: contribution of passive permeability to P-glycoprotein mediated efflux transport. Mol. Pharm. 2005, 2, 12–21. 10.1021/mp0499196. 15804173

[ref39] Gunturi S. B.; Narayanan R. In Silico ADME Modeling 3: Computational Models to Predict Human Intestinal Absorption Using Sphere Exclusion and kNN QSAR Methods. QSAR Comb. Sci. 2007, 26, 653–668. 10.1002/qsar.200630094.

[ref40] Moda T. L.; Torres L. G.; Carrara A. E.; Andricopulo A. D. PK/DB: database for pharmacokinetic properties and predictive in silico ADME models. Bioinformatics 2008, 24, 2270–2271. 10.1093/bioinformatics/btn415. 18684738

[ref41] Shen J.; Cheng F.; Xu Y.; Li W.; Tang Y. Estimation of ADME properties with substructure pattern recognition. J. Chem. Inf. Model. 2010, 50, 1034–1041. 10.1021/ci100104j. 20578727

[ref42] Guerra A.; Campillo N.; Paez J. Neural computational prediction of oral drug absorption based on CODES 2D descriptors. Eur. J. Med. Chem. 2010, 45, 930–940. 10.1016/j.ejmech.2009.11.034. 20022146

[ref43] sklearn.model_selection.train_test_split. https://scikit-learn.org/stable/modules/generated/sklearn.model_selection.train_test_split.html (accessed April 15, 2022).

[ref44] Moriwaki H.; Tian Y.-S.; Kawashita N.; Takagi T. Mordred: A molecular descriptor calculator. J. Cheminf. 2018, 10, 4 10.1186/s13321-018-0258-y. PMC5801138 29411163

[ref45] Chicco D.; Tötsch N.; Jurman G. The Matthews correlation coefficient (MCC) is more reliable than balanced accuracy, bookmaker informedness, and markedness in two-class confusion matrix evaluation. BioData Min. 2021, 14, 13 10.1186/s13040-021-00244-z. 33541410 PMC7863449

[ref46] AUC-ROC Curve in Machine Learning Clearly Explained. https://www.analyticsvidhya.com/blog/2020/06/auc-roc-curve-machine-learning/ (accessed May 4, 2022).

[ref47] Plonska A.; Plonski P. MLJAR: State-of-the-Art Automated Machine Learning Framework for Tabular Data, version 0.10.3. https://github.com/mljar/mljar-supervised (accessed 2021-04-15).

[ref48] Zurada J. M.; Malinowski A.; Cloete I. Sensitivity analysis for minimization of input data dimension for feedforward neural network. Proceedings of IEEE International Symposium on Circuits and Systems—ISCAS ’94; IEEE, 1994; Vol. 6, pp 447–450.

[ref49] Holzinger A.; Saranti A.; Molnar C.; Biecek P.; Samek W. Explainable AI Methods—A Brief Overview. xxAI—Beyond Explainable AI; Holzinger A., Goebel R., Fong R., Moon T., Müller K. R., Samek W., Eds.; Springer: Cham, 2022; Vol. 13200.

[ref50] Maschler M.; Solan E.; Zamir S. Game Theory; Cambridge University Press: Cambridge, 2013; Chapter 18 Shapley Values.

[ref51] Lundberg S.; Lee S. I. A Unified Approach to Interpreting Model Predictions. ArXiv. 2017, arXiv:1705.07874.

[ref52] Szlęk J. Model Interpretation. https://github.com/jszlek/MODEL_INTERPRETATION accessed (Nov 25, 2022).

[ref53] Getting Started with the RDKit in Python. https://www.rdkit.org/docs/GettingStartedInPython.html (accessed Nov 15, 2021).

[ref54] Abraham M. H.; Zhao Y. H.; Le J.; Hersey A.; Luscombe C. N.; Reynolds D. P.; Beck G.; Sherborne B.; Cooper I. On the mechanism of human intestinal absorption. Eur. J. Med. Chem. 2002, 37, 595–605. 10.1016/s0223-5234(02)01384-3. 12126778

