# Supplementary material for: Artificial Intelligence-Based Quantitative Structure–Property Relationship Model for Predicting Human Intestinal Absorption of Compounds with Serotonergic Activity
Source: Mol Pharm. 2023 Apr 18;20(5):2545–55. doi: 10.1021/acs.molpharmaceut.2c01117 (PMC10155205; doi:10.1021/acs.molpharmaceut.2c01117)
Supplement: Supplementary file 1 — mp2c01117_si_001.pdf [file mp2c01117_si_001.pdf]

Supporting Information

Artificial intelligence-based quantitative structure-property relationship model for predicting human intestinal absorption of compounds with serotonergic activity

Natalia Czub[1], Jakub Szlęk[1], Adam Pacławski[1*], Klaudia Klimończyk[1], Matteo Puccetti[2], Aleksander Mendyk[1]

[1]Department of Pharmaceutical Technology and Biopharmaceutics, Jagiellonian University Medical College, 30-688 Kraków, Poland

[2]Department of Pharmaceutical Sciences, University of Perugia, 06123 Perugia, Italy

Table 1. Summary of literature review with human intestinal absorption.

No.	Title	Authors	Year	Number of compounds with HIA
1.	Prediction of Human Intestinal Absorption of Drug Compounds from Molecular Structure [1]	Wessel, M. D., Jurs, P. C., Tolan, J. W., Muskal, S. M.	1998	86
2.	Physicochemical High Throughput Screening: Parallel Artificial Membrane Permeation Assay in the Description of Passive Absorption Processes [2]	Kansy, M., Senner, F., Gubernator, K.	1998	25
3.	Drug Liposome Partitioning as a Tool for the Prediction of Human Passive Intestinal Absorption [3]	Balon, K., Riebesehl, B. U., Müller, B. W.	1999	21
4.	Toward minimalistic modeling of oral drug absorption [4]	Oprea, T. I., Gottfries, J.	1999	85
5.	Molecular Hashkeys: A Novel Method for Molecular Characterization and Its Application for Predicting Important Pharmaceutical Properties of Molecules [5]	Ghuloum, A. M., Sage, C. R., Jain, A. N.	1999	20
6.	Rate-Limited Steps of Human Oral Absorption and QSAR Studies [6]	Zhao, Y. H., et al.	2002	237
7.	ADME evaluation: 2. A computer model for the prediction of intestinal absorption in humans [7]	Klopman, G., Stefan, L. R., Saiakhov, R. D.	2002	49
8.	Functional Role of P-Glycoprotein in Limiting Intestinal Absorption of Drugs: Contribution of Passive Permeability to P-Glycoprotein Mediated Efflux Transport [8]	Varma, M. V., Sateesh, K., Panchagnula, R.	2005	88
9.	*In Silico* ADME Modeling 3: Computational Models to PredictHuman Intestinal Absorption Using Sphere Exclusion and kNNQSAR Methods [9]	Gunturi, S.B., Narayanan, R.	2007	174
10.	PK/DB: database for pharmacokinetic properties and predictive in silico ADME models [10]	Moda, T. L., Torres, L. G., Carrara, A. E., Andricopulo, A. D.	2008	211
11.	Prediction of Human Intestinal Absorption by GA Feature Selection and Support Vector Machine Regression [11]	Yan, A., Wang, Z., & Cai, Z.	2008	518
12.	Estimation of ADME Properties with Substructure Pattern Recognition [12]	Shen, J., Cheng, F., Xu, Y., Li, W., Tang, Y.	2010	578
13.	Neural computational prediction of oral drug absorption based on CODES 2D descriptors [13]	Guerra, A., Campillo, N.E., Páez, J.A.	2010	201

14.	Selenocyanates and diselenides: A new class of potent antileishmanial agents [14]	Plano, D. et al.	2011	6
15.	Donepezil + propargylamine + 8-hydroxyquinoline hybrids as new multifunctional metal-chelators, ChE and MAO inhibitors for the potential treatment of Alzheimer's disease [15]	Wang, L. et al.	2014	9
16.	ChEMBL database [16] https://www.ebi.ac.uk/chembl/	-	Accessed May 2022	6

1. Wessel, M. D., Jurs, P. C., Tolan, J. W., & Muskal, S. M. (1998). Prediction of human intestinal absorption of drug compounds from molecular structure. *Journal of chemical information and computer sciences*, *38*(4), 726–735. https://doi.org/10.1021/ci980029a
2. Kansy, M., Senner, F., & Gubernator, K. (1998). Physicochemical high throughput screening: parallel artificial membrane permeation assay in the description of passive absorption processes. *Journal of medicinal chemistry*, *41*(7), 1007–1010. https://doi.org/10.1021/jm970530e
3. Balon, K., Riebesehl, B. U., & Müller, B. W. (1999). Drug liposome partitioning as a tool for the prediction of human passive intestinal absorption. *Pharmaceutical research*, *16*(6), 882–888. https://doi.org/10.1023/a:1018882221008
4. Oprea, T. I., & Gottfries, J. (1999). Toward minimalistic modeling of oral drug absorption. *Journal of molecular graphics & modelling*, *17*(5-6), 261–329. https://doi.org/10.1016/s1093-3263(99)00034-0
5. Ghuloum, A. M., Sage, C. R., & Jain, A. N. (1999). Molecular hashkeys: a novel method for molecular characterization and its application for predicting important pharmaceutical properties of molecules. *Journal of medicinal chemistry*, *42*(10), 1739–1748. https://doi.org/10.1021/jm980527a
6. Zhao, Y. H., Abraham, M. H., Le, J., Hersey, A., Luscombe, C. N., Beck, G., Sherborne, B., & Cooper, I. (2002). Rate-limited steps of human oral absorption and QSAR studies. *Pharmaceutical research*, *19*(10), 1446–1457. https://doi.org/10.1023/a:1020444330011
7. Klopman, G., Stefan, L. R., & Saiakhov, R. D. (2002). ADME evaluation. 2. A computer model for the prediction of intestinal absorption in humans. *European journal of pharmaceutical sciences : official journal of the European Federation for Pharmaceutical Sciences*, *17*(4-5), 253–263. https://doi.org/10.1016/s0928-0987(02)00219-1
8. Varma, M. V., Sateesh, K., & Panchagnula, R. (2005). Functional role of P-glycoprotein in limiting intestinal absorption of drugs: contribution of passive permeability to P-glycoprotein mediated efflux transport. *Molecular pharmaceutics*, *2*(1), 12–21. https://doi.org/10.1021/mp0499196
9. Gunturi, S.B., & Narayanan, R. (2007). In Silico ADME Modeling 3: Computational Models to Predict Human Intestinal Absorption Using Sphere Exclusion and kNN QSAR Methods. Qsar & Combinatorial Science, 26, 653-668. https://doi.org/10.1002/qsar.200630094
10. Moda, T. L., Torres, L. G., Carrara, A. E., & Andricopulo, A. D. (2008). PK/DB: database for pharmacokinetic properties and predictive in silico ADME models. *Bioinformatics (Oxford, England)*, *24*(19), 2270–2271. https://doi.org/10.1093/bioinformatics/btn415
11. Yan, A., Wang, Z., & Cai, Z. (2008). Prediction of human intestinal absorption by GA feature selection and support vector machine regression. *International journal of molecular sciences*, *9*(10), 1961–1976. https://doi.org/10.3390/ijms9101961
12. Shen, J., Cheng, F., Xu, Y., Li, W., & Tang, Y. (2010). Estimation of ADME properties with substructure pattern recognition. *Journal of chemical information and modeling*, *50*(6), 1034–1041. https://doi.org/10.1021/ci100104j
13. Guerra A, Campillo NE, Páez JA. Neural computational prediction of oral drug absorption based on CODES 2D descriptors. Eur J Med. Chem. 2010;45:930-940. https://doi.org/10.1016/j.ejmech.2009.11.034
14. Plano, D., Baquedano, Y., Moreno-Mateos, D., Font, M., Jiménez-Ruiz, A., Palop, J. A., & Sanmartín, C. (2011). Selenocyanates and diselenides: a new class of potent antileishmanial agents. *European journal of medicinal chemistry*, *46*(8), 3315–3323. https://doi.org/10.1016/j.ejmech.2011.04.054

15. Wang, L., Esteban, G., Ojima, M., Bautista-Aguilera, O. M., Inokuchi, T., Moraleda, I., Iriepa, I., Samadi, A., Youdim, M. B., Romero, A., Soriano, E., Herrero, R., Fernández Fernández, A. P., Ricardo-Martínez-Murillo, Marco-Contelles, J., & Unzeta, M. (2014). Donepezil + propargylamine + 8-hydroxyquinoline hybrids as new multifunctional metal-chelators, ChE and MAO inhibitors for the potential treatment of Alzheimer's disease. *European journal of medicinal chemistry*, *80*, 543–561. https://doi.org/10.1016/j.ejmech.2014.04.078
16. Mendez D., Gaulton A., Bento A.P., Chambers J., de Veij M., Félix E., Magariños M.P., Mosquera J.F., Mutowo P., Nowotka M., et al. ChEMBL: Towards direct deposition of bioassay data. Nucleic Acids Res. 2018;47:D930–D940. doi: 10.1093/nar/gky1075
